# Critical points of multiple defenses and system resilience reconstruction: a QCA configuration analysis and governance implications of maritime accidents

**DOI:** 10.3389/fpubh.2026.1809825

**Published:** 2026-07-06

**Authors:** Maosheng Li

**Affiliations:** Hainan Vocational University of Science and Technology, Haikou, China

**Keywords:** accident causation, governance modernization, maritime safety, Qualitative Comparative Analysis, system resilience

## Abstract

This study analyzes the complex causal mechanisms of maritime accidents in China's inland waterways. To capture conjunctural causation and equifinality, it employs Qualitative Comparative Analysis (QCA), integrating the “Swiss Cheese Model,” HFACS, and STAMP into a multidimensional framework of human, vessel, environmental, and management factors. Analysis of 33 real-world cases reveals that maritime safety is characterized by multiple conjunctural causation and causal asymmetry. Serious accidents are not triggered by single necessary conditions, though baseline compliance (e.g., proper routing and draft) appears as a trivial necessity. The study identifies six sufficient pathways to serious accidents, most notably “Resource-Constrained Nighttime Operational Failure”—where inadequate manning and nighttime conditions couple to overwhelm compliant operations—and “Systemic Safety Failure Driven by Economic Motives,” where overloading and manpower shortages driven by cost-cutting organizational decisions make accidents nearly inevitable. Contrasted with non-serious configurations highlighting “safety buffers,” findings show that while stressors like nighttime navigation multiply risk, robust internal compliance and resource redundancy effectively absorb them. Ultimately, the study advocates for a maritime governance paradigm shift from reactive response toward precision intervention and resilience reconstruction, providing a theoretical foundation for modern, adaptive maritime safety systems.

## 1 Introduction

Against the backdrop of increasingly frequent global trade and maritime activities, the operational efficiency and safety levels of maritime systems have become vital to national economic development and strategic security. As a major shipping nation, China has seen a continuous expansion in the scale of its maritime activities in recent years. In 2025, the maritime system effectively supported port operations involving 34.647 million vessel calls, 20.09 billion tons of cargo, and 310 million TEUs (Twenty-foot Equivalent Units), while ensuring the safe waterborne travel of 280 million passengers—with all indicators showing significant year-on-year growth (1).

Crucially, alongside this increase in transport volume, maritime traffic safety remained stable, with the number of accidents and fatalities decreasing by 36.7% and 13.8% year-on-year, respectively (2). This trend of “increased quantity with stabilized quality” signifies that China's maritime development has entered a new phase centered on high-quality development. Currently, the core objective for the maritime sector during the “15th Five-Year Plan” period has been defined as “accelerating the formation of a modern maritime supervision, service, and support system compatible with the construction of a leading transport nation” (3). To achieve this, maritime governance is undergoing a deep transformation along a developmental path characterized by “systematic improvement, intelligent leadership, legal safeguarding, and professional support” (4).

This transformation involves a multi-dimensional and multi-layered complex evolution. On one hand, the regulatory model is shifting from traditional reactive responses toward proactive prevention, enhancing governance effectiveness through institutional innovations such as the construction of a unified national credit management system. On the other hand, technical standards are iterating rapidly; the 2026 legislative agenda covers several frontier fields, including inland intelligent vessels, unmanned surface vehicles, and methanol/LPG-powered ships, reflecting a proactive response to green, low-carbon, and smart shipping trends (5). Concurrently, the construction of an integrated “land-sea-air-space” monitoring network and the application of artificial intelligence are driving the in-depth development of “Smart Maritime” services (6).

These interconnected policies, technologies, and regulatory measures collectively constitute a dynamically evolving complex system. While macro-level statistics indicate a generally stable and improving trend in China's maritime safety, it is crucial to emphasize that current statistical stability does not guarantee future system resilience. This trajectory of “quantitative growth coupled with qualitative stabilization” necessitates a deeper investigation into the increasingly complex coupling risks within the shipping system. Modern safety paradigms necessitate a shift from merely minimizing errors (Safety-I) toward strengthening the system's capacity to remain safe under varying conditions (Safety-II). In this view, investigating past accident causes is no longer about assigning blame to failing components but serves as a strategic foundation for resilience-based prevention. Consequently, the objective of this research adopts a dual-focus approach to dissect the systemic complexity of maritime safety under the pressure of high-intensity operations. The primary aim is to determine the non-linear disaster configurations (*Y*) formed by the interweaving of organizational management deficiencies, human resource bottlenecks, and complex environmental factors, while concurrently assessing the system's adaptive capacity by analyzing safety pathways (~*Y*). By contrasting these two pathways, this study provides analytical outcomes that facilitate a comprehensive shift in maritime governance—from reactive, post-accident responses to targeted, precision interventions and the proactive reconstruction of system resilience.

## 2 Literature review and methodology

As a typical socio-technical system, maritime safety performance does not depend on any single independent factor but originates from the dynamic coupling and interaction of subsystems, including humans, ships, environment, and management. Accidents are essentially chain-reaction failures of the system's defense architecture under multiple pressures—a perspective systematically expounded by James Reason (7). His “Swiss Cheese Model” vividly illustrates that systems possess multiple layers of defensive barriers, each with inherent flaws (or “holes”) (8). The “active failures” (such as operational errors) that directly trigger an accident often interact with “latent failures” deeply rooted in organizational decisions, management processes, and culture; an accident trajectory can only penetrate all barriers when the defects in each layer align perfectly in time and space (9).

Building upon this model, the Human Factors Analysis and Classification System (HFACS) established by Wiegmann and Shappell (10) provides a more operational analytical framework. HFACS categorizes accident causes into four levels of tiered influence: Organizational Influences, Unsafe Supervision, Preconditions for Unsafe Acts, and the Unsafe Acts themselves (11). This framework can be effectively integrated with the SHEL model (Software, Hardware, Environment, Liveware) proposed by Edwards (12), thereby systematically dissecting the vulnerable links of human-machine interaction within maritime systems.

Focusing on inland waterway shipping, systemic risks exhibit distinct contextual characteristics. According to the International Regulations for Preventing Collisions at Sea 1972 and China's Regulations on Administration of Traffic Safety on Inland Rivers (13), maintaining a “proper lookout” is both a statutory requirement and a core safety practice for crew members to establish and maintain situational awareness (14).

However, under the persistent pressure of latent failures—such as inadequate manning, accumulated fatigue, or nighttime/restricted visibility—an individual's cognitive defense capabilities are highly susceptible to weakening or total collapse. At this juncture, if these human factors undergo non-linear coupling with physical risks—such as vessel overloading, equipment hazards, or sudden adverse hydrological conditions—the system may rapidly breach the critical threshold of its overall resilience, leading to an accident.

Traditional statistical models, such as linear regression, primarily focus on estimating the “net effect” of individual variables on accident occurrence. This approach inherently relies on assumptions of causal linearity, symmetry, and the independence of variables. However, maritime accidents rarely follow such simplified patterns; instead, they typically manifest as non-linear collapses within complex systems. Consequently, linear methods struggle to capture the “conjunctural effects” of multiple risk factors interacting in specific contexts and often overlook “anomalous paths”—those configurations that may lack statistical significance across a large sample but possess catastrophic destructive power in reality.

In light of this causal complexity—characterized by high path dependency and equifinality (the principle that different combinations of causes can lead to the same outcome)—traditional linear thinking faces significant limitations in revealing how multiple conditions intersect. To address this gap, this study employs the QCA developed by Charles Ragin (15). Rooted in set theory and Boolean algebra, QCA treats each case as a unique configuration of antecedent conditions. Its core advantage lies in its ability to systematically navigate causal complexity through three dimensions: conjunction (how conditions combine to trigger outcomes), equifinality (identifying multiple pathways to the same accident), and causal asymmetry (recognizing that the configurations leading to failure are not simply the functional inverse of those ensuring safety) (16).

Traditional maritime accident research often falls into the dilemma of single-case analysis vs. purely quantitative statistical methods: the former offers depth but lacks generalizability, while the latter, though representative, relies on the logic of “Net Effects” and therefore has significant limitations in explaining the non-linear evolution of complex systems (17). Traditional linear regression models are usually based on the assumptions that independent variables are mutually independent and that causal relationships are linear and symmetric, making it difficult to capture the “Conjunctural Causation” effects of multiple risk factors in specific contexts (18). In fact, maritime accidents often exhibit “Equifinality,” meaning that distinctly different configurations of conditions can lead to the same accident outcome, and linear models tend to obscure these non-obvious disaster pathways (19). Hence, introducing QCA as a “third path” can not only effectively overcome the generalizability dilemma of single-case studies, but also reveal the complex, asymmetric interaction mechanisms among risk factors, thereby identifying the boundaries of system safety more precisely (20). It should be noted that this study is not merely a “retrospective tracing” of past maritime accidents. Instead, against the backdrop of a generally stable maritime safety environment, it aims to facilitate a paradigm shift in resilience governance from “reactive response” to “proactive defense.” The core logic of this research lies in dissecting the diverse configurational pathways of accidents to reveal the “critical points” essential for maintaining continuous system stability. By identifying these key thresholds, authorities can leverage redundant resources and precision interventions to reinforce systemic defenses before accident precursors manifest. This approach enables the long-term preservation of safety within dynamic and complex navigational environments, rather than passively waiting for accidents to occur before implementing remedial measures.

In recent years, cutting-edge research has increasingly integrated QCA with theoretical models that emphasize system control and interaction (21). For instance, the STAMP (Systems-Theoretic Accident Model and Processes) framework proposed by Leveson views safety as a dynamic control problem involving the flow of energy and information within a system (22). Its hierarchical control structure—spanning government, associations, management, operations, and processes—is highly complementary to the configurational logic of QCA (23).

This hybrid paradigm of “System Control Models + Configurational Comparative Analysis” has demonstrated powerful explanatory potential in accident analysis across high-risk industries such as chemical engineering, aviation, and energy (24). It enables a dual-dimensional perspective on accident generation mechanisms, examining both the vertical hierarchy (responsibility and control) and horizontal combinations (synergy of conditions) (25). In the domain of high-standard international safety, systemic accident models and configurational analysis methods have been thoroughly validated. In aviation, the STAMP-based CAST method has been adopted by NASA and the FAA for accident analysis and NextGen safety assessments, with empirical studies of airport incidents demonstrating that it yields more comprehensive safety recommendations (26). In the maritime domain, Nordic countries have pioneered practices (27): the Finnish VTS designed a safety management system based on STAMP and constructed an initial evaluation framework integrating Bayesian networks (28); Norway and Sweden have also applied STPA for risk assessment within the Sea Traffic Management project. Meanwhile, QCA, as a tool for uncovering causal complexity, has been widely applied in European public policy and complex risk management, for instance, the configurational comparison of COVID-19 lockdown policies across 26 European countries (29), and the identification of configurational pathways for cross-organizational collaborative governance in extreme disaster risk (30). These international experiences provide a solid practical reference and methodological foundation for this study's integration of STAMP control logic with QCA configurational thinking.

Applying this integrated framework to inland shipping safety research allows for condition calibration and configuration analysis of historical accident cases via csQCA, thereby identifying several typical paths leading to accidents. For instance, the analysis might reveal causal patterns with inherent logical consistency, such as “Organizational Resource Scarcity + Lack of Supervision + Nighttime Environmental Stress” or “Culture of Non-compliant Operation + Insufficient Vessel Seaworthiness + Sudden Natural Forces.” This configuration-based insight can drive maritime regulation to shift from reactive punishment targeting isolated violations toward precision intervention based on systemic risk path warnings. Consequently, it facilitates a governance paradigm shift from post-accident accountability to ex-ante system resilience reinforcement, providing solid theoretical support and methodological tools for constructing a more forward-looking and adaptive inland shipping safety governance system.

To frame the precise mechanism through which system resilience fractures, this study introduces the concept of “Critical Points of Multiple Defenses,” capturing the specific configurational state in complex socio-technical systems where such breakdowns occur. In the context of the Swiss Cheese Model (SCM), this not only means that the “holes” in different defense layers align accidentally in time and space, forming a failure trajectory that penetrates all barriers; more importantly, by incorporating the STAMP (Systems-Theoretic Accident Model and Processes) perspective, this study clarifies that “defenses” should not be understood merely as physical barriers (e.g., life-saving equipment or hull structures), but rather as dynamic “control mechanisms.” These mechanisms encompass organizational decisions, operational constraints, and feedback loops. Therefore, the “Critical Points of Multiple Defenses” are essentially the non-linear coupling point where physical defense failures and systemic control constraint collapses converge. When these configurational conditions are jointly satisfied, the system loses its capacity to absorb risk, making accidents inevitable.

Regarding the research paradigm, the exploration of “system resilience reconstruction” in this study is grounded in the Safety-II theoretical framework. In accordance with Hollnagel (31), resilience should not be viewed as merely “repairing damaged components” but rather as improving the system's ability to adapt (31). Therefore, the examination of non-accident pathways (~*Y*) in this research acts as an empirical illustration of how the system effectively adjusts and maintains safety amidst operational challenges (31). By conducting a comparative study of “safety buffer” configurations in non-serious accident cases (~*Y*), this research reveals the resilience mechanisms that allow systems to maintain stable operations despite perturbations such as nighttime operational pressure or inadequate manning (31).

To framework the precise mechanism through which system resilience fractures, this study unifies SCM and STAMP by conceptualizing “Critical Points of Multiple Defenses,” preventing these theories from operating independently. In this study, “Critical Points” are characterized as the theoretical juncture where structural weaknesses (illustrated by latent gaps in the Swiss Cheese Model's defensive layers) coincide with a failure in systemic control mechanisms (as outlined by STAMP's dynamic constraints and feedback loops). This integration underscores that the alignment of “holes” is not a stochastic accident but a logical consequence of control failures. Such a unified framework supports the application of QCA to analyze the specific configurations in which defensive penetrations and systemic control collapses intersect, thereby revealing the non-linear dynamics of maritime accident causation.

In summary, this study proposes the following core proposition: Inland waterway accidents are not mere aggregations of factors, but results of non-linear coupling between SCM and STAMP. Because such dual collapses manifest significant configurational complexity and causal asymmetry—which conventional linear methods fail to capture—it is theoretically necessary to employ QCA to identify the synergistic pathways driving complex accidents and support system resilience.

## 3 Research design

### 3.1 Methodological framework

This study employs csQCA. Crisp-set analysis treats conditions and outcomes as binary variables (0 or 1), requiring researchers to assign cases to explicit membership categories. Grounded in set theory and Boolean algebra, this method views cases as configurations of conditions and aims to identify the necessary and sufficient conditions leading to an outcome through cross-case comparison. Unlike traditional statistical methods that focus on correlations between variables, csQCA examines the set-theoretic relationships between combinations of conditions and outcomes (20). It is particularly suitable for research problems where conditions are distinct and cases can be clearly categorized. Its core assumptions include:

(1) Asymmetry: The combinations of conditions that lead to a severe accident (Y = 1) are not simply the logical opposite of those that prevent one (Y = 0). For example, this study finds that the combination of “inadequate manning, nighttime navigation, and operational error” may lead to an accident; however, this does not imply that “adequate manning, daytime navigation, and error-free operation” will necessarily guarantee safety. Safety may be secured by an entirely different set of configurations. In this methodological context, analyzing the configuration of ~Y is not merely a mathematical exercise but a deliberate shift toward the Safety-II perspective, focusing on the conditions under which maritime systems successfully absorb risks;
(2) Equifinality: Multiple distinct causal paths can lead to the same severe outcome. This analysis identifies six sufficient paths leading to severe accidents (e.g., resource-constrained types, economically-driven types, etc.), verifying the “multiple causes, same effect” characteristic of maritime system failures from various perspectives;
(3) Conjunctural Causation (or Conjunction): The effect of a single condition is not fixed; rather, it is highly dependent on the specific combination of conditions in which it situated. For instance, “nighttime navigation” may have its risks buffered by system resilience when combined with “adequate manning and compliant operation.” However, when combined with “inadequate manning” or “overloading,” it easily becomes a critical catalyst that amplifies risk and triggers an accident.

Furthermore, this study achieves a methodological synergy between the Swiss Cheese Model (SCM) and STAMP through QCA. Although they rest on different ontological foundations—SCM focuses on static defensive failures, while STAMP emphasizes dynamic control deficiencies—the two are complementary when viewed through the lens of system resilience. Within the framework of this study, SCM serves to delineate the “physical boundaries” of the safety system, determining the initial conditional variables for QCA by identifying the holes in defensive layers such as personnel, vessels, and environment. STAMP, in turn, provides the “logical nexus,” explaining how the failure of control constraints at the management level leads to non-linear coupling among these defensive layers. By analyzing the configurational relationships between these “defensive holes” and “control failures,” the QCA method integrates originally isolated barrier failures into a systemic control logic, thereby revealing the compound mechanism of maritime accidents that spans from defense penetration to control collapse. Specifically, each model serves a distinct function in the procedure: SCM defines defense boundaries and QCA antecedents; HFACS acts as an operational tool for feature extraction and Coding; and STAMP is employed for Interaction Analysis to explain the non-linear coupling of systemic failures.

The overall research process is illustrated in the figure below:

(See Figure 1).

**Figure 1:**
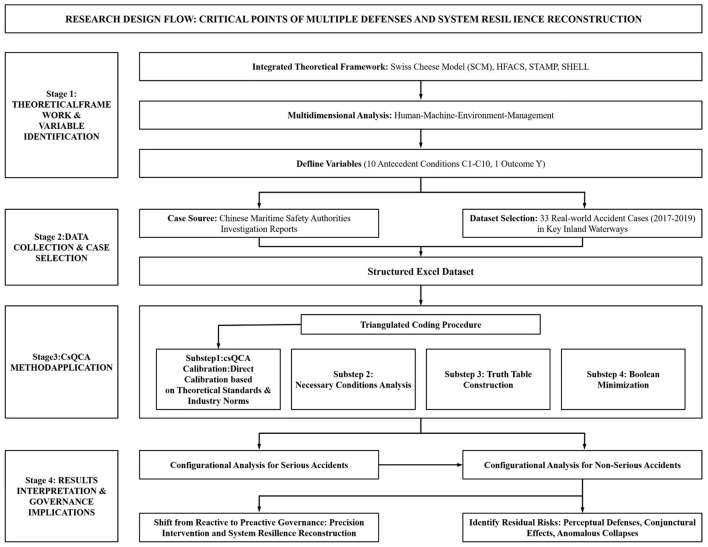
Methodological roadmap.

### 3.2 Data sources

To ensure theoretical sampling transparency—a critical requirement for the validity of QCA—the 33 cases were purposefully selected through a rigorous multi-stage filtering process. Initially, a broader preliminary dataset was constructed drawing upon official investigation reports from the China Maritime Safety Administration (MSA) and statistical databases from the Shanghai Ship and Shipping Research Institute (SSSRI). The raw data underwent an ETL (Extract, Transform, Load) standardization process to systematically capture 15 key fields, including vessel routing, hydrological/meteorological conditions, and accident sequences.

To align precisely with this study's focus on the causal complexity of commercial inland waterway systems, strict inclusion and exclusion criteria were applied: (1) accidents involving non-standard commercial transport, such as fishing vessels and tugboats, were excluded due to their fundamentally different operational risk profiles (32); (2) incidents exclusively driven by pilotage errors were removed to accurately isolate crew-level behaviors and systemic organizational defects. Furthermore, unlike traditional linear regression which relies on large-N statistical significance, QCA requires “thick descriptions” to identify conjunctural causation. Therefore, the final screening retained only 33 highly representative cases from 2017 to 2019 that possessed complete, deeply detailed causal chain records. These 33 cases are not limited to a single accident type but encompass a diverse range of typical inland incidents (e.g., collisions, groundings, and founderings), providing a highly focused, information-rich empirical foundation for configurational analysis without compromising necessary diversity.

Its core design aims to transform rich qualitative text (e.g., detailed accident narratives) and quantitative information (e.g., manning numbers, cargo volume) into variables suitable for analysis. Through explicit coding rules, 10 binary conditional variables—such as “Inadequate Manning,” “Improper Operation,” “Vessel Failure,” and “Failure to Follow Designated Routes”—as well as status variables like “Nighttime Occurrence” and “Overloading” can be extracted. This fully satisfies the requirements of the csQCA method for crisp-set processing.

Furthermore, the structured numerical and categorical fields provide a foundation for exploring correlations between variables (e.g., the correlation between manning status and operational errors). The cases exhibit sufficient diversity in terms of accident types (collision, grounding, and foundering, etc.) and vessel types, ensuring the representativeness and comparative value of the sample. In summary, with its structured information presentation, potential for objective variable transformation, and excellent adaptability to multivariate analytical methods, this dataset provides a solid and reliable empirical foundation for systematically exploring the configurational causal paths and internal correlations of concurrent factors in maritime accidents.

It is acknowledged that this study faces the challenge of limited diversity, a common issue in QCA research when the number of antecedent conditions (*k* = 10) yields a large property space (2^10^ = 1024) relative to the number of observed cases (*N* = 33). However, this design is justified by the nature of maritime accidents as complex, rare events where empirical observations are naturally clustered in specific high-risk configurations. To mitigate the impact of limited diversity, the analysis follows the standard minimization procedure and focuses on the “intermediate solutions,” which incorporate only theoretically plausible counterfactuals. The results represent the observed causal complexity within the existing accident reports rather than an exhaustive mapping of all logical possibilities.

### 3.3 Construction and calibration of conditions and outcome variables

Based on accident causation theory and a review of existing literature, this study identifies 10 antecedent condition variables (C1–C10) and one outcome variable (*Y*). The specific definitions are detailed in Table 1 below.

**Table 1:** Definition of conditions, outcome variables, and calibration anchors.

Variable code	Variable name	Assignment criteria (calibration)
C1	Inadequate manning	1 = Actual crew < Minimum manning requirement; 0 = Otherwise
C2	Vessel failure	1 = Presence of steering failure, hull damage, engine room failure, short circuit, etc.; 0 = None
C3	Improper operation	1 = Errors in turning, berthing, loading, or navigation; 0 = None
C4	Failure to follow designated routes	1 = Taking the wrong channel, deviating from route, or violating navigation rules; 0 = None
C5	Overloading	1 = Loaded tonnage > Deadweight tonnage; 0 = Not overloaded
C6	Improper draft	1 = Excessive draft or exceeding maximum allowable draft; 0 = Normal
C7	Lookout negligence	1 = Failure to maintain a proper lookout or late detection of obstacles/vessels; 0 = None
C8	Nighttime/Restricted visibility	1 = Occurred between 22:00–06:00 or during poor visibility; 0 = Daytime/Good visibility
C9	Adverse weather/Hydrological conditions	1 = Presence of strong winds, rapid currents, rising waters, etc.; 0 = None
C10	Terminal/Fairway facility issues	1 = Unsafe terminal facilities, missing buoys, or lack of construction warnings; 0 = None
Y	Accident severity	1 = Severe accident; 0 = Minor accident

This study adopts the direct assignment method, utilizing fsQCA software to assign values to the 11 variables of each case based on the aforementioned anchors, thereby converting original “Yes/No” or categorical data into binary membership scores of 0 (non-membership) or 1 (full membership). For example, the outcome variable Y was calibrated based on established severity standards: cases involving “no casualties, no foundering, and no obstruction to navigation” were assigned a score of 0, while those involving “minor injuries, foundering, or obstruction to navigation,” as well as “fatalities, missing persons, or major losses,” were unified and calibrated as 1.

#### 3.3.1 Human factors dimension

##### 3.3.1.1 C1: Inadequate manning

This variable evaluates if actual crew size meets statutory Minimum Safe Manning Standards. These regulations ensure sufficient expertise for navigation and emergency response; falling below these levels compromises safety redundancy and increases fatigue. In high-pressure scenarios, manpower shortages delay critical actions and elevate accident risks. By comparing required vs. actual crew numbers from accident reports, this study applies an objective binary (0/1) assignment. Including “Inadequate Manning” quantifies human resource gaps as managerial defects and examines their contribution to maritime accidents.

##### 3.3.1.2 C3: Improper operation

This variable identifies crew decision-making or execution errors during navigation and operations. Maritime accidents often stem from operational breaks, such as misjudged course changes or incorrect cargo stowage, which are linked to skill, situational awareness, and procedural adherence. By analyzing accident reports for specific triggers like “grounding during a turn,” this study isolates “Improper Operation” as an independent variable. This allows for a clear assessment of how human error, training effectiveness, and compliance serve as key precursors to accidents.

##### 3.3.1.3 C4: Failure to follow designated routes

This variable identifies vessel violations of established navigation rules and channel regulations. While inland systems use beacons and traffic separation schemes to organize flow, deviating from designated routes or entering prohibited areas increases grounding and collision risks. Such behavior often stems from equipment failure, missing updates, or intentional shortcuts. By extracting explicit report evidence like “entered the wrong channel,” this study distinguishes “Violation of Navigation Rules” from general operational errors. It focuses specifically on navigational discipline and compliance, reflecting the vessel's overall standardization and risk assessment capabilities.

##### 3.3.1.4 C7: Lookout negligence

This variable measures a core cause of collisions: the failure to maintain a proper lookout as mandated by COLREGs 1972 (33). It requires using sight, hearing, and all available technology to continuously assess collision risks. Lookout negligence leads to delayed detection of hazards, resulting in late or non-existent evasive actions—the most common direct trigger for accidents. In this study's collision cases, “failure to maintain a proper lookout” is frequently cited as a decisive factor. By isolating this variable, the framework addresses the situational awareness and risk identification stages, quantifying how perception failures—driven by distraction, over-reliance on aids, or poor watchkeeping—combine with other conditions to cause accidents.

#### 3.3.2 Vessel status dimension

##### 3.3.2.1 C2: Vessel failure

This variable identifies accidents triggered by hardware failure or loss of hull integrity. A vessel's seaworthiness relies on subsystems like propulsion, steering, and electrical power; failures in these areas can lead to loss of control, fires, or foundering. Such incidents often stem from underlying maintenance gaps, aging, or inspection defects. Since accident reports frequently cite “steering gear failure” or “hull breach” as event triggers, this variable represents the technical and material deficiencies involved (34). It effectively links technical management standards with overall safety outcomes.

##### 3.3.2.2 C5: Overloading

This variable quantifies the risks of exceeding a vessel's statutory load line for commercial gain. Overloading breaches legal safety boundaries based on stability and structural strength, reducing reserve buoyancy and increasing the likelihood of capsizing or structural failure. This study uses case data (e.g., actual tonnage vs. legal limits) to provide objective evidence of non-compliance. By designating “Overloading” as an independent variable, the framework highlights how economic-driven decisions and a poor safety culture serve as critical accident precursors. It further examines how overloading interacts with other factors, such as degraded maneuverability, to exacerbate accident severity.

##### 3.3.2.3 C6: Improper draft

This variable characterizes technical risks stemming from a mismatch between actual vessel draft and available channel depth (35). “Over-draft” navigation occurs when a vessel exceeds depth restrictions due to seasonal changes or human activity, significantly increasing the risk of grounding, hull damage, and cargo shifting. By comparing reported draft against waterway depth notices, this variable identifies instances where draft exceeds physical constraints. It often reflects deeper management failures, such as poor voyage planning, risky commercial decision-making, or inaccurate loading calculations.

#### 3.3.3 Environmental conditions dimension

##### 3.3.3.1 C8: Occurrence during nighttime or restricted visibility

This variable operationalizes external stress factors, specifically time and visibility, at the moment of an accident. Nighttime navigation and restricted visibility (fog, rain, or snow) significantly impair visual lookouts and judgment while increasing crew fatigue and cognitive decline. Statistics confirm that accident rates and severity peak during these unfavorable periods. In this study, the variable is assigned a value of 1 if the accident occurred between 22:00 and 06:00 or during documented restricted visibility. This objective design allows for an analysis of whether accidents cluster under poor sensory conditions and how these external factors amplify existing human or vessel deficiencies.

##### 3.3.3.2 C9: Adverse weather / hydrological conditions

This variable identifies the role of natural environmental forces, such as strong winds, dense fog, rapid currents, or abnormal tides, in the accident causal chain. These factors can impair maneuverability, reduce visibility, or alter channel conditions. The variable is assigned when accident reports explicitly cite elements like “strong winds” or “rising water” as key factors. It captures extreme physical challenges that serve as primary causes or external stressors. As a core component of the “Environment-Human-Vessel” interaction model, this variable helps analyze how environmental conditions couple with human error or equipment failure to trigger or exacerbate maritime accidents (36).

#### 3.3.4 Management and external environment dimension

##### 3.3.4.1 C10: Terminal/fairway facility issues

This variable identifies external safety support failures—such as inaccurate navigation aids, poor terminal facilities, or inadequate construction warnings—that fall beyond a vessel's control. By analyzing cases like unnotified construction cables or poorly secured shore-side hulls, this variable (C10) measures the reliability of maritime public safety services. It shifts the focus from isolated ship errors to systemic failures within the broader safety management network, offering policy insights for industrial supervision.

To ensure methodological rigor, the 10 antecedent conditions were calibrated using the direct method based on industry norms and theoretical standards. A triangulated coding procedure was employed, where two researchers independently assigned binary values (0/1) to the 33 accident reports. With an initial intercoder agreement of 93%, discrepancies were resolved through consensus with a senior maritime safety expert. This rigorous, systematic approach ensures the transformation from qualitative narratives to data is transparent, stable, and replicable.

#### 3.3.5 Setting the degree of incident impact

This study sets Accident Severity (*Y*) as the outcome variable, categorized by casualties, vessel damage, and navigational impact. Y = 0 represents minor accidents with no casualties, sinking, or obstruction to traffic. Y = 1 includes serious and severe accidents involving injuries, fatalities, missing persons, or major property loss (such as capsizing or sinking). This classification reflects the core goals of maritime safety—protecting life and maintaining navigational order—while accounting for social and economic impacts. These clear criteria allow for consistent data extraction from accident reports, meeting the QCA method's requirement for observable variables. This approach helps identify the causal configurations that lead to different severity levels.

### 3.4 Data analysis

Following data calibration, the analysis proceeds in three stages. First, a necessity analysis is conducted on the conditional variables to determine whether a specific variable must be present for an outcome to occur (37). Generally, a condition is regarded as necessary if its Consistency metric exceeds 0.9 (38). In this study, tests will be conducted separately for Y = 0 (minor accidents) and Y = 1 (non-minor accidents) to identify key conditions that almost always precede a specific outcome. Second, a Truth Table is constructed to aggregate the data. Each row in the table represents a logically possible combination of conditions, indicating the number of corresponding cases and the resulting consistency. Finally, Boolean minimization is performed (39). The Truth Table undergoes standardized analysis using Boolean algebraic algorithms to simplify the combinations leading to the presence or absence of the outcome. This process generates complex, parsimonious, and intermediate solutions. This study will primarily report and interpret the intermediate solutions, incorporating “logical remainders” where theoretically and logically justifiable to derive more substantively meaningful causal configurations (40).

Furthermore, it should be acknowledged that the empirical necessity of certain negated conditions (e.g., ~C4 and ~C6) is likely an artifact of limited diversity inherent in the 33-case sample. In the context of inland waterway navigation, “not deviating from course” and “normal draft” represent ubiquitous baseline compliance states; their high consistency scores in this study reflect the empirical distribution of the sampled reports rather than a universal causal law. The primary contribution of this research, therefore, lies not in the identification of single-factor necessity, but in uncovering the six sufficient configurational pathways that trigger serious accidents. This approach shifts the focus from isolated variables to the complex, non-linear coupling of multiple defenses, providing a more granular understanding of system resilience and failure.

To ensure the rigor and reproducibility of the configurational analysis in this study, the case frequency threshold was set at 1, the raw consistency threshold at 0.8, and the PRI (Proportional Reduction in Inconsistency) consistency threshold was maintained above 0.7. During the process of Boolean minimization, this study reports the intermediate solution. This choice is justified as it incorporates only those counterfactual reminders that align with theoretical expectations of maritime traffic accidents, thereby generating accident path configurations with greater explanatory power.

The necessity analysis for serious accidents (*Y*) reveals that the negated conditions ~C4 and ~C6 exhibit a consistency of 1.000. However, necessity should not be interpreted solely based on consistency. An examination of their low coverage scores (0.322 and 0.312, respectively) and Relevance of Necessity indicates that they are trivial necessary conditions. In this dataset, “not deviating from course” and “normal draft” are ubiquitous baseline compliance states. Because they are present in the vast majority of cases (both Y and ~*Y*), they are empirically consistent but lack the theoretical relevance to be considered active causal drivers. Consequently, no single positive causal factor constitutes a theoretically relevant necessary condition for serious accidents.

Overall, ~C4, ~C6, ~C9, and ~C10 demonstrate extremely high consistency across both types of accident outcomes, reflecting their status as foundational baseline conditions (trivial necessities) rather than discriminatory causal triggers. Since necessity analysis only reflects the constraint of single conditions and cannot reveal synergistic effects between variables, the subsequent research will further explore the causal configurations (intermediate solutions) leading to different accident outcomes by constructing a Truth Table and performing Boolean minimization to obtain more substantively significant empirical findings. In the configurational analysis, a frequency threshold of 1 was adopted, which is methodologically appropriate for a medium-N sample size of 33 to retain maximum empirical information. The raw consistency threshold was set at 0.9, significantly exceeding the recommended minimum of 0.75, while the Proportional Reduction in Inconsistency (PRI) consistency was maintained above 0.7 to minimize logical contradictions and ensure the validity of the identified sufficient combinations (Table 2).

**Table 2:** Analysis of necessary conditions.

Outcome	Outcome variable: ***Y***		Outcome variable: ~***Y***	
Conditions tested	Consistency	Coverage	Consistency	Coverage
C1	0.800000	0.444444	0.434783	0.555556
~C1	0.200000	0.133333	0.565217	0.866667
C2	0.200000	0.250000	0.260870	0.750000
~C2	0.800000	0.320000	0.739130	0.680000
C3	0.400000	0.500000	0.173913	0.500000
~C3	0.600000	0.240000	0.826087	0.760000
C4	0.000000	0.000000	0.086957	1.000000
~C4	1.000000	0.322581	0.913043	0.677419
C5	0.300000	0.750000	0.043478	0.250000
~C5	0.700000	0.241379	0.956522	0.758621
C6	0.000000	0.000000	0.043478	1.000000
~C6	1.000000	0.312500	0.956522	0.687500
C7	0.500000	0.500000	0.217391	0.500000
~C7	0.500000	0.217391	0.782609	0.782609
C8	0.800000	0.333333	0.695652	0.666667
~C8	0.200000	0.222222	0.304348	0.777778
C9	0.100000	1.000000	0.000000	0.000000
~C9	0.900000	0.281250	1.000000	0.718750
C10	0.100000	0.500000	0.043478	0.500000
~C10	0.900000	0.290323	0.956522	0.709677

#### 3.4.1 Conditional configuration analysis

Through configurational analysis, this study determined that the overall solution coverage for the occurrence of the outcome is 0.7, indicating that the identified combinations of conditions can explain approximately 70% of the relevant cases. Furthermore, both the overall solution consistency and the consistency of each specific pathway exceed the 0.75 threshold. This demonstrates that these configurational relationships possess high reliability and explanatory power, robustly reflecting the causal patterns observed within the cases.

(1) Configurations for Serious Accidents (*Y*)
(2) Configurations for Non-Serious Accidents (~*Y*)

In the configurational analysis for non-serious accidents (~*Y*), the resulting overall solution coverage is 0.913043. This indicates that the multiple identified combinations of conditions collectively explain approximately 91.3% of non-serious accident cases, demonstrating high explanatory power (41). Furthermore, both the overall solution consistency and the consistency of each individual pathway exceed 0.75, with most pathways achieving a consistency of 1. This indicates that these configurational relationships possess high robustness and reliability within the empirical cases, satisfying the fundamental quality requirements for sufficient condition combinations in QCA.

The results clearly and robustly support the core hypothesis of the study: inland maritime accidents are not triggered by a single, universal necessary cause, but are the result of multiple antecedent conditions acting together in specific configurations. The necessity analysis reveals that although individual conditions show high consistency, no single condition stands out as the sole, distinctive driver of outcomes. Instead, whether leading to serious accidents (*Y*) or non-serious accidents (~*Y*), these individual conditions represent pervasive systemic factors rather than isolated triggers. This finding deeply corroborates the core concept of the “Swiss Cheese Model”—that accidents stem from the accidental alignment of holes in multiple defensive layers across time and space, rather than the complete failure of a single layer—thereby negating the possibility of simple attribution at the systemic level.

The configurational analysis further reveals “multiple pathways to accidents.” For serious accidents (*Y*), the intermediate solutions identify six distinct sufficient combinations of conditions, with an overall solution consistency of 1 (perfect) and a coverage of 0.7. This indicates that these six pathways can explain approximately 70% of serious accident cases, and each pathway serves as a sufficient means of leading to an accident.

In contrast, non-serious accidents (~*Y*) also present seven sufficient pathways (consistency of 0.9545, coverage of 0.913), featuring combinations of conditions that differ significantly from those leading to serious accidents. This provides preliminary confirmation of the principle of causal asymmetry in QCA (Tables 3, 4). Through the visual representation of “system interaction” and “risk coupling,” each configuration is specifically demonstrated as a unique “risk recipe,” clearly revealing how human factors, vessel status, environmental conditions, and external management factors couple and interact non-linearly to collectively overwhelm the safety resilience of the system.

**Table 3:** Configurations for serious accidents.

Conditional variables	H1	H2	H3	H4	H5	H6
C1	❖	❖	⊚	❖	❖	❖
C2	⊚	⊚	❖	⊚	❖	⊚
C3	❖	❖	⊚	⊚	❖	⊚
C4	⊚	⊚	⊚	⊚	⊚	⊚
C5		❖	❖	⊚	⊚	⊚
C6	⊚	⊚	⊚	⊚	⊚	⊚
C7	⊚		⊚	❖	❖	⊚
C8	❖	❖	❖	❖	⊚	❖
C9	⊚	⊚	⊚	⊚	⊚	❖
C10	⊚	⊚	⊚	⊚	⊚	❖
Raw coverage	0.2	0.2	0.1	0.1	0.1	0.1
Unique coverage	0.1	0.1	0.1	0.1	0.1	0.1
Consistency	1	1	1	1	1	1
Overall solution coverage	0.7					
Overall solution consistency	1					

In the table above, ❖ indicates the presence of a core condition, ❖ indicates the presence of a peripheral condition, ⊚ indicates the absence of a core condition, ⊚ indicates the absence of a peripheral condition, and an empty cell indicates that the presence or absence of the variable is irrelevant.

**Table 4:** Configurations for non-serious accidents (~*Y*).

Conditional variables	NH1	NH2	NH6	NH3	NH4	NH5	NH7
C1		⊚	⊚	⊚	❖	⊚	⊚
C2		⊚	⊚	⊚	⊚	⊚	⊚
C3	⊚	❖	⊚	⊚	⊚	⊚	❖
C4	⊚	⊚	⊚	❖	⊚	⊚	⊚
C5	⊚	⊚	⊚	⊚		⊚	⊚
C6	⊚	⊚	⊚	⊚	⊚	❖	⊚
C7	⊚		❖	⊚	⊚	⊚	❖
C8	❖	⊚	⊚		❖	⊚	⊚
C9	⊚	⊚	⊚	⊚	⊚	⊚	⊚
C10	⊚	⊚	❖	⊚	⊚	⊚	⊚
Raw coverage	0.521739	0.130435	0.0434783	0.0869565	0.217391	0.0434783	0.0434783
Unique coverage	0.347826	0.130435	0.0434783	0.0869566	0.0434783	0.0434783	0.0434783
Consistency	0.923077	1	1	1	0.833333	1	1
Overall solution coverage	0.913043						
Overall solution consistency	0.954545						

The symbols carry the same meanings as defined above.

The following section provides a detailed interpretation of the six configurations within the intermediate solutions, translating the Boolean expressions into “risk narratives” that hold practical significance for maritime safety management (42):

Path 1:C1^*^~C2^*^C3^*^~C4^*^~C6^*^~C7^*^C8^*^~C9^*^~C10

In the configurational analysis of accident causation, this path represents a typical evolutionary pattern of “Resource-Constrained Nighttime Operational Failure.” This path reveals how deep-seated systemic vulnerabilities trigger accidents through the coupling of “Human-Environment-Machine” factors (43), even when the objective physical environment and navigational conditions appear to be fundamentally compliant.

Using the SHELL model, this path focuses on the gap between Software (manning policies), Environment (nighttime/restricted visibility), and Liveware (crew operations). First, Inadequate Manning (C1) creates systemic hazards by increasing fatigue and the cognitive load on the remaining crew. Second, during high-risk periods of Nighttime or Restricted Visibility (C8), the crew's visual perception is limited, which may impair their ability to process complex information.

According to the Swiss Cheese Model, although the vessel's hardware is in good condition (~C2), route planning is scientific (~C4), and external meteorological or facility conditions are normal (~C9, ~C10)—meaning multiple defensive barriers in the system appear intact—the organizational defense failure caused by manning shortages resonates with environmental pressures during specific time periods. This causes an instantaneous collapse of otherwise robust operational procedures, which eventually translates into specific Improper Operations (C3). This phenomenon of “surface-level compliance but deep-seated vulnerability” demonstrates that in maritime safety management, simple hardware maintenance and the fulfillment of static regulations (~C4, ~C6, ~C7) cannot fully offset the negative effects triggered by Inadequate Manning (C1) under extreme environments (C8). This finding underscores the necessity of Proactive Safety Management: the redundancy of human resource allocation and operational control during high-risk periods must be treated as critical system safety variables, rather than relying solely on the safety of the physical environment.

Path 2: C1^*^~C2^*^C3^*^~C4^*^C5^*^~C6^*^C8^*^~C9^*^~C10

This path presents a form of “Systemic Safety Failure Driven by Economic Motives.” It not only inherits the negative impacts of human resource shortages and environmental pressures from Path 1 but also introduces the core variable of “Overloading (C5).” This elevates the risk from a simple “operational-level failure” to a systemic collapse under the triple constraints of “Physical-Psychological- Organizational” factors.

From the perspective of Trajectory Intersection Theory, this path reveals the intertwining of unsafe human acts (C3) and unsafe states of objects (C5) within a specific temporal and spatial environment (C8). Overloading (C5) is not merely a legal violation; from the standpoint of ship dynamics, it drastically compresses the ship's stability margin and delays maneuverability response. This creates a dangerous “risk resonance” with the lack of cognitive resources among the crew caused by Inadequate Manning (C1). Under the complex working conditions of Nighttime or Restricted Visibility (C8), the crew is already in a state of limited perception. The unstable and sluggish operational feedback of an overloaded vessel further exacerbates the crew's psychological pressure and operational misjudgments, ultimately leading to the complete penetration of defensive barriers.

Analyzed through Accident Chain Theory, this configuration reflects a shipping enterprise's systemic disregard for safety boundaries in the pursuit of maximizing economic interests. Inadequate manning (C1) and overloading (C5) often share a common organizational root cause: sacrificing organizational resilience to cut costs. This operating model traps the vessel in a dangerous “high-load, low-guarantee” zone. Despite compliant routing (~C4) and fair weather conditions (~C9, ~C10), the strong coupling effects triggered by organizational management defects reduce the system's fault tolerance to nearly zero. This “upgraded” path demonstrates that when illegal loading andmanpower shortages work in synergy, it is more likely to lead to serious accidents. It profoundly reflects the severe consequences of the imbalance between economic benefits and safety bottom lines in maritime safety.

Path 3: ~C1^*^C2^*^~C3^*^~C4^*^C5^*^~C6^*^~C7^*^C8^*^~C9^*^~C10

This path presents a specific evolutionary logic characterized by “Technical Failure and the Breach of Physical Boundaries.” The core feature of this configuration is that even when Human Factors are relatively robust—manifested as adequate manning (~C1), error-free operation (~C3), and diligent lookout (~C7)—accidents still occur due to the penetration of the vessel system's underlying physical barriers. From the perspective of System Safety Theory, this confirms that the compensatory capacity of human effort against systemic risks is limited.

According to the Energy Unexpected Release Theory, Overloading (C5) essentially increases the difficulty of controlling a vessel's kinetic and gravitational potential energy while simultaneously weakening its stability reserves under adverse conditions. In this context, the occurrence of Critical Equipment Failure (C2) acts as the “trigger” for the uncontrolled release of energy. This state of “operating with defects and violations,” catalyzed by the specific environmental factor of Nighttime (C8), causes the vessel to completely lose its System Resilience. Although routing is compliant (~C4) and personnel are dutiful, the physical performance degradation caused by overloading and equipment failure produces a strong negative synergy, directly leading to the loss of dynamic equilibrium.

This path profoundly reveals the dialectical relationship between “Hard Safety” and “Soft Safety” in maritime security. It emphasizes the insurmountable nature of vessel technical conditions (C2) and loading compliance (C5) as physical safety boundaries. In a complex “Human-Machine-Environment” system, if the “Machine” (the vessel itself), which serves as the material foundation, possesses systemic defects and illegal loads, then even if the “Human” element exerts its maximum defensive effectiveness, it cannot break the accident chain triggered by the collapse of physical limits. This conclusion powerfully demonstrates that maintaining the Intrinsic Safety of a vessel is the cornerstone of navigational safety; any breach of the physical bottom line (such as overloading or sailing with mechanical defects) will cause the system's risk defense mechanism to fall into a holistic failure due to a lack of physical support.

Path 4: C1^*^~C2^*^~C3^*^~C4^*^~C5^*^~C6^*^C7^*^C8^*^~C9^*^~C10

This path presents an evolutionary trajectory characterized by the “Prior Failure of the Perceptual Hierarchy.” The core logic of this configuration is that even when the vessel is in a seaworthy state (~C2), loading and draft are compliant (~C5, ~C6), and there is no direct operational error (~C3), the system collapse originates at the most fundamental defensive outpost: Lookout Negligence (C7). Analyzed through Endsley's Situation Awareness Model, Inadequate Manning (C1) constitutes a chronic organizational stressor (44). This stress triggers a “fatigue resonance” during Nighttime/Restricted Visibility (C8) (45), a period when physiological and perceptual capabilities are naturally weakened. At this point, a fracture occurs in the crew's extraction of environmental information (Level 1 SA), leading to an inability to correctly comprehend and project potential collision risks, thereby forming a typical “perceptual defense black hole.”

According to Man-Machine-Environment System Theory, a lookout is not merely a physical activity but a dynamic monitoring process highly dependent on cognitive resources. The shortage of personnel (C1) forces on-duty staff to extend watchkeeping hours or undertake multi-tasking, drastically depriving them of the cognitive redundancy required to maintain high-intensity attention. During the night (C8), when external reference points are scarce, crew members easily fall into a state of “sensory deprivation” or “monotony fatigue,” making Lookout Negligence (C7) the first link to break in the system's defensive chain. Even if the voyage plan is scientific (~C4) and meteorological conditions are stable (~C9), the failure of this first line of safety reduces the system's ability to detect external sudden risks (such as illegal maneuvers by other vessels) to zero.

This path profoundly demonstrates the decisive role of human resource allocation and circadian rhythm management in navigational safety. It warns management that safety depends not only on visible “hardware compliance” but also on invisible “perceptual continuity.” Lookout negligence (C7) is not an isolated human error but a systemic symptom catalyzed by a lack of organizational resources and high-risk environmental pressures. Consequently, prevention for such paths must move beyond simple disciplinary action for violations. Instead, it must focus on reinforcing the foundation of situational awareness—by optimizing watchkeeping schedules, introducing fatigue monitoring technologies, and strengthening nighttime auxiliary detection methods (such as infrared/radar-linked alarms)—to prevent catastrophic consequences caused by a break in the perceptual chain.

Path 5: C1^*^C2^*^C3^*^~C4^*^~C5^*^~C6^*^C7^*^~C8^*^~C9^*^~C10

This configuration presents a typical evolutionary pattern of “Non-Environment- Dependent Systemic Collapse.” The core logic of this path is that even when external extreme triggers—such as nighttime visibility restrictions (~C8), overloading (~C5), and adverse weather (~C9)—are excluded, the system still plunges into disaster due to the simultaneous penetration of multiple internal defensive barriers. Analyzed through Perrow's Normal Accident Theory (46), this reflects the Tight Coupling relationship between internal elements within modern vessels as highly complex socio-technical systems. Inadequate Manning (C1) and Equipment Failure (C2), acting as pre-existing organizational and technical defects, unexpectedly trigger a chain reaction of Lookout Negligence (C7) and Improper Operation (C3) during the routine working conditions of daylight, possibly due to a “defensive relaxation” in the crew's psychological alertness.

According to Man-Machine-Environment System Engineering Theory, although environmental factors (~C8) provide a better perceptual baseline for the system, they cannot compensate for the underlying absence of organizational management (C1) and hardware reliability (C2) (47). When management deficiencies coexist with technical failures, the system enters an extremely fragile period of equilibrium. At this point, any minor human deviation (C3, C7) is rapidly amplified through internal system interactions. This “multi-point resonance” phenomenon validates a contemporary evolution of Heinrich's Domino Theory (48): accidents no longer stem solely from the fall of a single “domino,” (49) but from the dynamic convergence of organizational defects (Management), technical status (Machine), and behavioral failure (Human) in a specific time and space, which pierces all remaining safety redundancies (50).

This path serves as a profound warning against the “illusion of environmental dependency” in maritime safety management. The findings of Path 5 demonstrate that the collapse of a safety bottom line often stems from the latent serial connection of systemic weaknesses. Even during the day when objective navigational conditions are superior, if human resource allocation (C1) cannot support equipment maintenance and standard watchkeeping operations, the system's fault tolerance will be extremely compressed. Therefore, improving Intrinsic Safety should not focus solely on responding to extreme environments (51). Instead, it must start from repairing the baseline of the core risk triangle—“Manning-Equipment-Behavior”—to eliminate the systemic drivers that allow multiple minor risks to aggregate into a catastrophic accident (52).

Path 6: C1^*^~C2^*^~C3^*^~C4^*^~C5^*^~C6^*^~C7^*^C8^*^C9^*^C10

This path reveals a typical disaster pattern characterized by “Extreme Environmental Coupling and Lack of Organizational Redundancy.” The logic of this path suggests that when a system is confronted with a triple external threat— comprising Nighttime (C8), Adverse Weather/Hydrological Conditions (C9), and External Infrastructure Defects (C10)—the occurrence of an accident no longer depends on a single individual error, but rather on the overall Robustness of the system against disturbances. From the perspective of System Resilience Theory, Inadequate Manning (C1) does not manifest here as a direct operational fault; instead, it represents the exhaustion of the system's “Safety Margin.” (53) Under extreme working conditions, sufficient human resources should have served as a “buffer” to handle sudden environmental variables. However, the presence of C1 causes the system to lose its necessary dynamic adjustment capabilities and fault-tolerant space when faced with the dual pressure of C9 and C10.

According to the High Reliability Organization theory, the key for complex systems to cope with extreme risks lies in the detection of and rapid response to “weak signals” (54). Although the crew in this path maintained a normal lookout (~C7) and committed no active operational errors (~C3), the information entropy of the system increased sharply under the superimposed intervention of nighttime adverse weather and navigational facility failures (55). Its complexity exceeded the cognitive boundaries that a streamlined manning level (C1) could handle. This confirms an extreme case in Barrier Defense Theory: even if all human defensive barriers (Soft Barriers) are in place, the system will still undergo an irreversible collapse if the external attack load (Environmental Load) exceeds the structural strength of both the physical and organizational components (56).

This path demonstrates that maritime safety involves more than just the vessel; it is an open system that interacts with the environment and infrastructure. The findings of Path 6 suggest that under extreme environmental warnings (C9), the reliability of external safety support (C10) is a critical safeguard. Additionally, shipping enterprises should note that adequate human resources (~C1) are not just for daily operations, but serve as a safety redundancy to maintain defenses during external shocks. This support helps shift safety management from “human error control” toward “system resilience enhancement.” QCA reveals both how accidents are caused and how serious consequences are avoided. Analyzing non-serious accident configurations (~*Y*) provides a perspective on a system's safety buffers and resilience. The analysis shows that pathways leading to ~Y (with 91.3% solution coverage) differ from those leading to Y. This supports the principle of causal asymmetry in QCA—that safety is not just the opposite of an accident, but is formed by a unique set of condition combinations.

The study finds that the most representative safety pathway (~C3^*^~C4^*^~C5^*^~C6^*^~C7^*^C8^*^~C9^*^~C10, covering 52.2% of cases) exhibits a core characteristic: resisting “external pressure” through rigorous “internal compliance.” Although the incident occurs during nighttime or periods of restricted visibility (C8)—a typical external environmental stressor—the vessel maintains high levels of standardization and integrity across multiple dimensions, including human operations (no improper operation ~C3, no routing violations ~C4, no lookout negligence ~C7), vessel status (no overloading ~C5, normal draft ~C6), and external support (no adverse weather ~C9, no facility issues ~C10). This configuration vividly illustrates the positive side of the “Swiss Cheese Model”: when defensive barriers at the organizational and technical levels (corresponding to the “Preconditions for Unsafe Acts” and “Unsafe Acts” levels in the HFACS framework) remain solid and intact (57), even if a “hole” appears at the environmental level (C8), the “trajectory” of the accident can only penetrate limited layers (58). Its energy is effectively buffered and contained within the range of minor consequences. This provides empirical evidence that strict adherence to navigational regulations and operating procedures serves as the most reliable cornerstone of resilience for a system to withstand uncertainty and achieve “damage control” (59).

Compared to the multiple and complex defect-coupling pathways leading to serious accidents (*Y*), the pathways for ~Y exhibit a more “pure” risk logic. Other safety pathways—such as the combination of “adequate manning and no nighttime navigation” (~C1^*^~C8) or “no critical equipment failure and compliant operations”—all point to the same conclusion: the core of avoiding serious consequences lies in preventing the simultaneous failure of multiple key risk dimensions, especially proactive human errors and technical hardware defects. This stands in sharp contrast to the “multiple-defect superposition” pattern commonly found in the pathways leading to Y (60). For instance, in Y-related pathways, nighttime (C8) often co-occurs with factors like inadequate manning (C1), improper operation (C3), or vessel failure (C2), creating a “stress-defect resonance” effect. Conversely, in ~Y-related pathways, nighttime is frequently accompanied by compliant conditions such as “no improper operation” and “no overloading,” transforming its role from an “accident amplifier” into a “manageable routine risk” (61).

First, nighttime navigation (C8) is inherently “context-dependent” (62). Rather than being a decisive disaster-causing factor, it serves as a risk-sensitivity multiplier. Its ultimate role is to amplify the effects of other conditions with which it is combined: when coupled with internal defects, it catalyzes major catastrophes; when coupled with internal compliance, its risks are absorbed by the system's fault tolerance. This transcends the limitations of linear thinking—which simply categorizes nighttime as a high-risk factor—and demands that management practices shift from the crude logic of “prohibiting nighttime navigation” toward a strategy of precision control that “ensures absolute reliability in other dimensions during nighttime navigation” (63).

Secondly, adequate manning (~C1) demonstrates a powerful systemic buffering function. While it appears as a critical defect (C1) in most pathways leading to Y, it emerges as a protective condition in multiple pathways leading to ~Y. This strongly suggests that adequate manning is more than just a baseline regulatory requirement; it provides the redundancy and adaptive capacity necessary for the system to handle emergencies, correct accidental errors, and distribute workloads. It serves as the core “resource reserve” for enhancing overall system resilience.

Finally, proactive violations or errors (such as C3 improper operation, C5 overloading, and C7 lookout negligence) demonstrate a stronger correlation with accident severity. These factors are generally absent from the primary pathways leading to ~Y, yet they appear with high frequency in the pathways leading to Y. This indicates that, compared to technical failures or environmental mutations that are difficult to avoid entirely, “behavioral risks” triggered by proactive human choices or negligence often serve as the more direct and lethal “final blow” that leads to catastrophic losses (64). This provides clear guidance for the priority allocation of safety management resources: curbing proactive violations and enhancing operational reliability should be placed at the core of risk prevention and control.

The analysis of configurations for non-serious accidents not only validates the value of maintaining the integrity of multiple defensive layers from a reverse perspective but also points the way for the development of safety management systems from the philosophical height of “causal asymmetry” (65). It suggests that safety governance should shift from the unattainable goal of “zero defects”toward the construction of asymmetric resilience advantages (66). This means ensuring continuous compliance in critical dimensions—such as eliminating proactive errors and guaranteeing resource adequacy—to weaken or offset the impact of environmental or technical uncertainties that are difficult to eradicate. This aligns with the concept in System Safety Engineering Theory that views safety as a “dynamic control process” (67). It pushes maritime regulation to undergo a fundamental paradigm shift: moving from passive response to accidents toward proactively shaping anti-fragile systems—elastic systems capable of containing consequences within an acceptable range even under extreme pressure.

In light of the risk mechanisms revealed by the aforementioned configurational paths, maritime policymakers should move away from a “one-size-fits-all” general regulatory approach and instead prioritize interventions at the highly sensitive coupling point of “Human Resources–Spatio-Temporal Environment.” Research indicates that manning deficiencies (C1) and lookout negligence (C7) are the core triggers leading to the collapse of system resilience during nighttime (C8) and adverse weather (C9). Consequently, the primary intervention should focus on establishing a “Dynamic Manning Compensation Mechanism.” Utilizing the “Land-Sea-Air-Space” integrated monitoring system (such as the linkage between AIS and VTS), regulators can identify vessels in a “low manning, high workload” state in real-time. Before such vessels enter nighttime periods or complex navigation segments, shore-based monitoring centers should mandate intervention via VTS voice verification, infrared-assisted detection, or satellite remote sensing alerts. These technical means manually supplement the system's “perceptual redundancy” and “decision-making buffer.” Such a strategy can precisely break the fatigue and cognitive failure chains described in Path 1 and Path 4, curbing the chain reactions triggered by insufficient system resilience at the source.

Secondly, another critical intervention point should be anchored in “Hard Constraints on Physical Boundary Compliance” to block systemic failures driven by economic incentives (Paths 2 and 3). Given that overloading (C5) and operating with defective equipment (C2) are often the decisive factors that escalate accident consequences from “ordinary” to “severe,” policy tools should shift from traditional post-incident inspections to sensor-based “off-site precision supervision.” Maritime authorities should prioritize the deployment of automated draft identification systems and ship energy efficiency monitoring platforms at inland water hubs, major ports, and key channel points, deeply linking real-time loading data with the credit management system. For vessels identified within high-risk configurations (e.g., overloaded with a history of steering gear failures), “geofencing” navigation restrictions should be enforced. Furthermore, these vessels should be included in the priority supervision list of the “Double Random and One Open” system. By constructing a rigorous “Risk + Credit” dual defense framework, policymakers can ensure vessels consistently operate within safe physical stability limits, thereby eliminating the institutional risks associated with overextending system safety margins for short-term economic gains.

#### 3.4.2 Residual risks: systemic challenges beyond traditional frameworks and the urgency of this study

Although the number of maritime traffic accidents and fatalities in China has shown an overall downward trend, with the safety situation continuously improving, this study argues that beneath this macro-level trend of “quantitative growth and qualitative improvement” lies a type of “residual risk” that cannot be ignored. Specifically, these are systemic or anomalous accidents arising from non-linear coupling within complex systems, which fall outside the risk domains covered by existing regulatory rules and technical standards. The six severe accident paths identified in this paper, particularly the “economy-driven systemic safety failure” (Path 2) and the “coupling of extreme environments with a lack of organizational redundancy” (Path 6), precisely illustrate the typical forms of such risks.

Examined from the theoretical perspective of risk governance, “residual risk” does not imply that the risk remains unidentified; rather, it indicates that under the current governance paradigm, these risks are difficult to eliminate merely through the reinforcement of single rules. Their characteristics manifest in at least three dimensions. First, the hidden nature of causality. Path 4, “premature failure at the perceptual level,” demonstrates that under conditions of vessel hardware compliance and seemingly normal operations, the mere coupling of fatigue and a nighttime environment can lead to the silent collapse of the “first line of defense”—lookout negligence. Such defects are extremely difficult to detect during traditional compliance inspections, yet they constitute the very source of systemic vulnerability. Second, the conjunctural nature of causation. Path 1, “resource-constrained nighttime operational errors,” reveals that inadequate manning does not act in isolation; instead, it generates a conjunctural effect of “simultaneous penetration of multiple defensive layers” when combined with nighttime environments and operational errors. This implies that merely targeting a single violation (such as penalizing inadequate manning) while ignoring its coupling with environmental and human factors makes it fundamentally difficult to disrupt the accident chain. Third, the anomalous nature of risks. In Path 6, an accident occurs while the vessel is operating normally with no violations, triggered by the triple superposition of extreme weather, navigational infrastructure defects, and inadequate manning. This type of “abnormal collapse under normal conditions” is precisely the sort of “Normal Accident” that traditional linear thinking struggles to explain.

The existence of these residual risks endows this study with its core focus and urgency: as the safety situation continues to improve, the marginal decline in accident rates does not equate to the completion of governance tasks. Instead, it compels us to shift our focus from “frequent violations” to “low-frequency, high-consequence” systemic anomalies, and from the control of “known risks” to the identification of and defense against “unknown couplings.” Essentially, this is precisely the ontological advantage of the QCA method: it excels at capturing “multiple conjunctural causation” and “causal asymmetry,” allowing those configurational paths—which are often overlooked in statistical models due to their rarity yet are sufficient to trigger severe consequences in reality—to become visible. In this sense, what conventional rules can prevent is often merely the recurrence of historical precedents; the true test of the modernization of maritime governance lies in its capacity to perceive those unilluminated areas within the system's defensive architecture before “residual risks” can evolve into disasters.

## 4 Conclusion

This study employs the csQCA method to systematically analyze the complex causal mechanisms of maritime accidents in China's inland waterways, revealing the multiple conjunctural causal nature of maritime safety as a typical socio-technical system (68). The findings indicate that accidents do not stem from a single necessary condition; rather, they are the result of the coupling of multiple antecedent conditions—such as human factors, vessel status, environmental pressures, and management defects—in specific configurations. The research identifies six sufficient pathways leading to serious accidents, including “Resource-Constrained Nighttime Operational Failure” (69), “Systemic Safety Failure Driven by Economic Motives” (70), and “Extreme Environmental Coupling and Lack of Organizational Redundancy” (71). Each pathway embodies the logic of spatial and temporal alignment of holes in multiple defensive layers as described in the Swiss Cheese Model, as well as the dynamic process of energy and information control failure within STAMP (22). This discovery fundamentally negates a linear view of safety based on simple attribution, corroborating the non-linear, emergent, and path-dependent nature of maritime safety. It provides an empirical basis rooted in configurational thinking for understanding the underlying question of “why accidents happen” (72).

From the perspective of theoretical construction, this study integrates the HFACS human factors framework (73), the SHEL model, and the STAMP system theory to establish a multidimensional analytical framework characterized by “vertical hierarchical control + horizontal conditional synergy.” This approach allows maritime accident analysis to transcend the limitations of traditional attribution.

The analysis shows that the same external condition, such as nighttime navigation, plays different roles depending on the configuration. When combined with management or human defects—like inadequate manning or operational errors—it acts as a risk amplifier. However, when paired with compliant operations and vessel seaworthiness, its risks are often absorbed by system resilience. This illustrates the “causal asymmetry” in QCA and reflects the importance of “redundant resources” in safety theory. Therefore, safety governance should not necessarily pursue absolute “zero risk.” Instead, it should focus on building resilience by using consistent compliance in key areas to neutralize unavoidable uncertainties.

Based on these conclusions, this study offers insights for modernizing maritime governance. Regulation should transition from “reactive punishment” for isolated violations to “precision intervention” based on systemic risk warnings. In practice, “reconstructing system resilience” requires shifting the governance focus from penalizing isolated violations to implementing precision interventions based on the identified failure configurations. Specifically, to counteract resource-constrained configurations (e.g., Paths 1 and 4), resilience reconstruction demands implementing dynamic human resource compensation and cognitive assistance (such as reinforced watchkeeping or radar-linked alarms) during high-stress periods like nighttime, thereby enhancing the system's absorptive resilience against environmental shocks. For configurations driven by economic motives or technical failures (e.g., Paths 2 and 3), regulatory technologies must strictly enforce physical boundaries like load lines and vessel seaworthiness, building a rigid boundary resilience capable of withstanding potential human operational errors. Finally, to mitigate the superimposed impact of extreme environments and organizational redundancy deficits (Path 6), resilience must be reconstructed by breaking the isolation of “single-vessel defense”; this involves strengthening the “vessel-shore-management” collaborative network—such as providing reliable fairway infrastructure and real-time hydrological alerts—to forge collaborative restorative resilience. Through this configuration-based intervention, maritime safety management can genuinely transition from reactive post-accident accountability to the proactive cultivation of systemic anti-fragility. This approach provides practical pathways for a modern maritime regulatory system that aligns with national development goals.

## Data Availability

The original contributions presented in the study are included in the article/supplementary material, further inquiries can be directed to the corresponding author.
